# Supplementary material for: Subnanometer-resolution structure determination in situ by hybrid subtomogram averaging - single particle cryo-EM
Source: Nat Commun. 2020 Jul 24;11:3709. doi: 10.1038/s41467-020-17466-0 (PMC7381653; doi:10.1038/s41467-020-17466-0)
Supplement: Supplementary file 1 — Supplementary Information [file 41467_2020_17466_MOESM1_ESM.pdf]

Supplementary Information for

Subnanometer-resolution structure determination *in situ* by a hybrid subtomogram averaging - single particle cryoEM - workflow

Ricardo Sanchez, Yingyi Zhang, Wenbo Chen, Lea Dietrich, Misha Kudryashev#

# corresponding author, email: misha.kudryashev@biophys.mpg.de

Supplementary Figures and Figure Legends

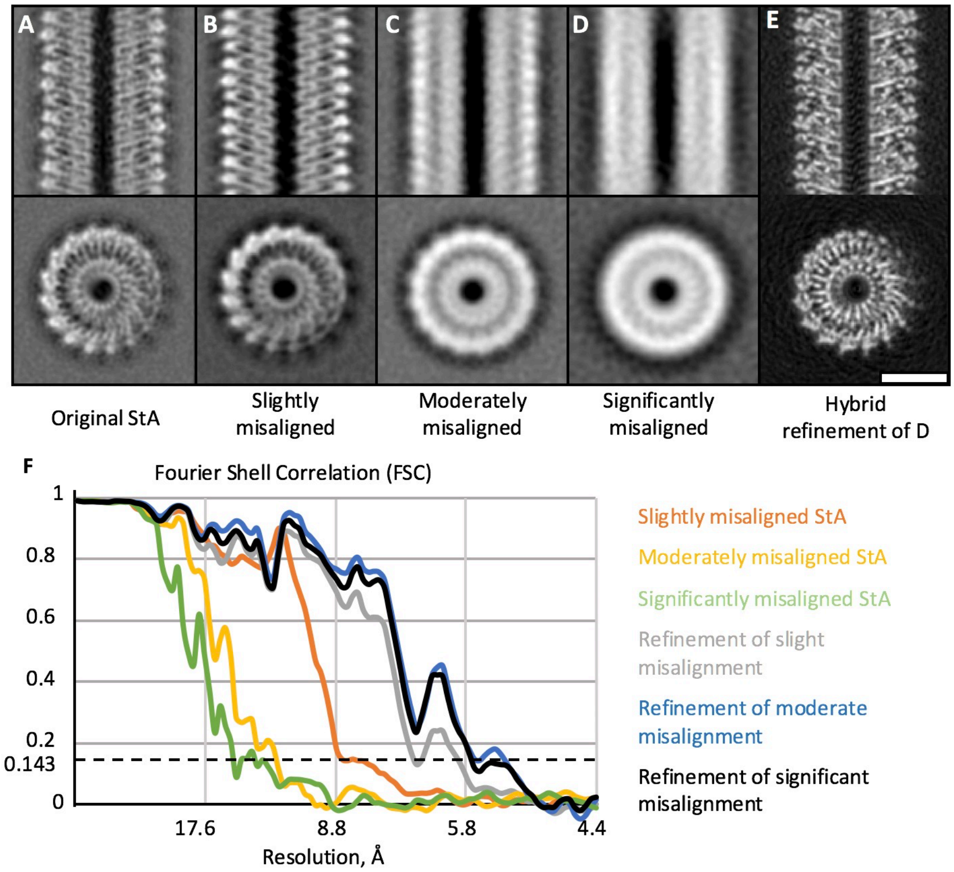

Supplementary Figure 1. Recovering high-resolution information from the misaligned TMV dataset using hybrid StA refinement. The shifts and rotations for the initial alignment (in A) were perturbed slightly (B), moderately (C) or significantly (D) which reduced the resolution. Hybrid refinement recovered the resolution to similar to the original levels (E, F). Scale bar: 10 nm.

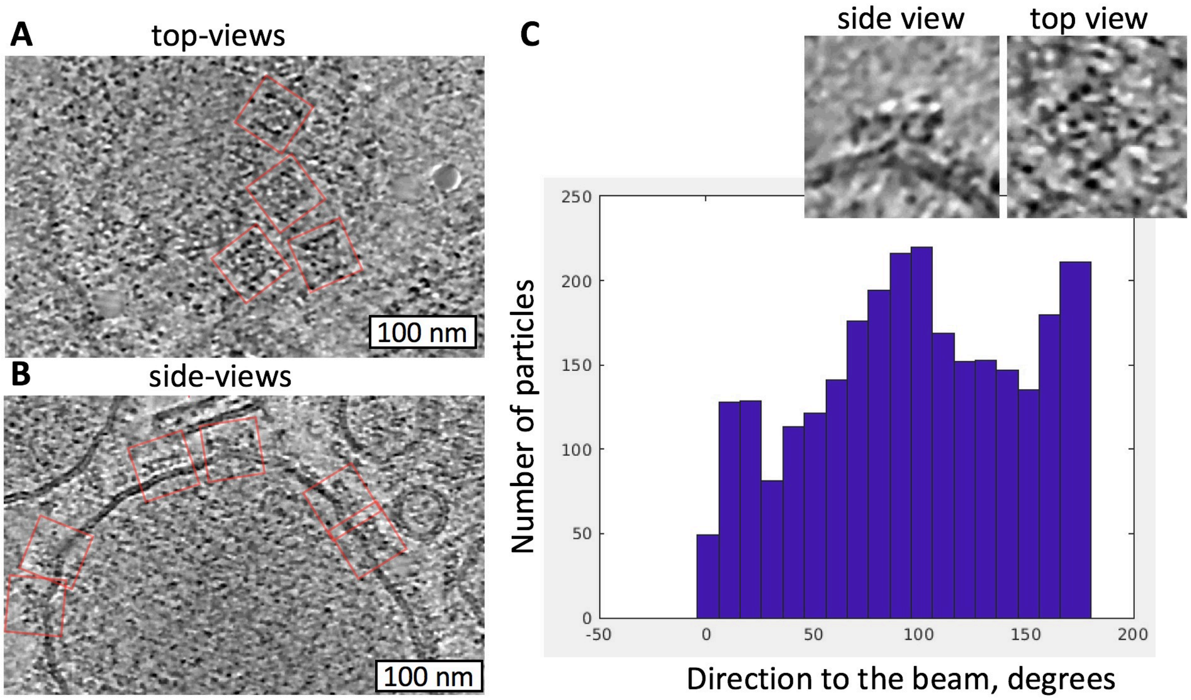

Supplementary Figure 2. Distribution of orientations for RyR1. A, B) Slices through tomograms of native SR vesicles with the top- and side-views of RyR1 indicated by red boxes. C) Distribution of the angles to the electron beam for the RyR1 dataset with 10 degree bins. Zero and 180 degrees correspond to top- and bottom-views, 90 degree corresponds to views from the sides.

Supplementary Table 1. Details for cryo-EM data collection

	TMV (EMD10834)<br><br>hStA map without CTF refinement	RyR1 (EMD 10840)<br><br>hStA map
Electron microscope	Thermo Fisher Krios G2 with a post-GIF K2 (Gatan)	Thermo Fisher Krios G1 with a post-GIF K2 (Gatan)
Magnification	64000	81000
Voltage (kV)	300	300
Electron exposure (e–/Å^2)	15	16
Defocus range (µm)	-3…-3.5	-3.5 … -4.5
Pixel size (Å)	2.2	1.7
Symmetry imposed	helical	c4
Final particle images (no.)	20,214	2,563
Map resolution (Å)	5,2	9,1
FSC threshold	0.143 with independent subsets	0.143 with independent subsets
Original data	EMPIAR-10393	EMPIAR-10452

Supplementary Note 1

```
MacroName HybridDoseSymmetricTomo
# Adopted from the script of Wim J.H.Hagen (EMBL Heidelberg 2015)
# Roll Buffers A-> H.
# Uses LowDose
# Run eucentric rough and fine
# Track plus K
# Track min L
# Record plus M
# Record min N

########## SETTINGS ##########

step            = 3     # stage tilt step in degrees
tilttimes       = 10    # multiply by 4 images + 1 image
Tiltbacklash    = -3    # negative tilts will be backlashed, must be negative value!

Driftcrit       = 3     # Angstrom/second
Driftinterval   = 10    # wait time between drift measurements in seconds
Drifttimes      = 5     # maximum number of drift measurements before skipping

########## END SETTINGS ##########


ResetClock
echo =====> batchrun HybridDoseSymmetricTomo

tiltangle = 0

CallFunction HybridDoseSymmetricTomo::TiltZero

# prevent runaway focus
AbsoluteFocusLimits -10 10
FocusChangeLimits -2 2

Loop $tilttimes
        # tilt plus1
tiltangle = $tiltangle + $step
CallFunction HybridDoseSymmetricTomo::TiltPlus
        # tilt min1
tiltangle = -1 * $tiltangle
CallFunction HybridDoseSymmetricTomo::TiltMinus
        # tilt min2
tiltangle = $tiltangle - $step
CallFunction HybridDoseSymmetricTomo::TiltMinus
        # tilt plus2
tiltangle = -1 * $tiltangle
CallFunction HybridDoseSymmetricTomo::TiltPlus
EndLoop

TiltTo 0
```

```
ResetImageShift
SetDefocus 0
echo =======================================

function TiltZero
# store stage position
ReportStageXYZ
StageX = $ReportedValue1
StageY = $ReportedValue2

# drift and tracking
T
Copy A K
Copy A L
Delay $driftinterval
Loop $drifttimes index
          T
          AlignTo K
                ReportAlignShift
          dx = $reportedValue3
          dy = $reportedValue4
          dist = sqrt $dx * $dx + $dy * $dy
          rate = $dist / $driftinterval * 10
          echo Rate = $rate A/sec
          If $rate < $driftcrit
                    echo Drift is low enough after shot $index
                    break
          Elseif $index < $drifttimes
                    Delay $driftinterval
          Else
                    echo Drift never got below $driftcrit: Skipping …
                    break
          Endif
EndLoop

# autofocus
G
G
G

# store defocus
ReportDefocus
focusplus = $RepVal1
focusmin = $RepVal1

# acquire the "high-dose" tilt image here
```
Set Exposure R 15
```
R
S
RestoreCameraSet
Copy A M
```

```
Copy A N

# store image shifts
ReportImageShift
ISxplus = $RepVal1
ISyplus = $RepVal2
ISxminus = $RepVal1
ISyminus = $RepVal2

## tracking after just to be sure
#T
#Copy A K
#Copy A L
endfunction

function TiltPlus
# tilt stage
TiltTo $tiltangle

# reset stage XY
MoveStageTo $StageX $StageY

# set defocus and image shift
GoToLowDoseArea R
SetDefocus $focusplus
SetImageShift $ISxplus $ISyplus

# drift and tracking
T
AlignTo K
Delay $driftinterval
Loop $drifttimes index
        T
        AlignTo K
        ReportAlignShift
        dx = $reportedValue3
        dy = $reportedValue4
        dist = sqrt $dx * $dx + $dy * $dy
        rate = $dist / $driftinterval * 10
        echo Rate = $rate A/sec
        If $rate < $driftcrit
                    echo Drift is low enough after shot $index
                break
        Elseif $index < $drifttimes
                Delay $driftinterval
        Else
                echo Drift never got below $driftcrit: Skipping …
                break
        Endif
EndLoop
```

```
# autofocus. Two rounds. Remove one G for single focus round.
G
G

# store defocus
ReportDefocus
focusplus = $RepVal1

# acquire tilt image
R
S

# tracking after
AlignTo M
Copy A M

# store image shifts
ReportImageShift
ISxplus = $RepVal1
ISyplus = $RepVal2

# new track reference
T
Copy A K
endfunction

Function TiltMinus
# tilt stage with backlash
TiltTo $tiltangle
TiltBy $Tiltbacklash
TiltTo $tiltangle

# reset stage XY
MoveStageTo $StageX $StageY

# set defocus and image shift
GoToLowDoseArea R
SetDefocus $focusmin
SetImageShift $ISxminus $ISyminus

# drift and tracking
T
AlignTo L
Delay $driftinterval
Loop $drifttimes index
	T
	AlignTo L
	ReportAlignShift
	dx = $reportedValue3
	dy = $reportedValue4
	dist = sqrt $dx * $dx + $dy * $dy
```

```
	rate = $dist / $driftinterval * 10
	echo Rate = $rate A/sec
	If $rate < $driftcrit
		echo Drift is low enough after shot $index
		break
	Elseif $index < $drifttimes
		Delay $driftinterval
	Else
		echo Drift never got below $driftcrit: Skipping …
		  break
	Endif
EndLoop

# autofocus. Two rounds. Remove one G for single focus round.
G
G

# store defocus
ReportDefocus
focusmin = $RepVal1

# acquire tilt image
R
S

# tracking after
AlignTo N
Copy A N

# store image shifts
ReportImageShift
ISxminus = $RepVal1
ISyminus = $RepVal2

# new track reference
T
Copy A L
endfunction
```

Supplementary Note 2

The script below could be used together with the built-in Tilt Series functionality of SerialEM. It modifies the exposure times for high- and low-dose images and, importantly, overwrites the parameters specified in the SerialEM setup. In case of strong preferred orientation TargetHighDoseAngle could be modified, in this case dose-symmetric checkbox probably should be disabled in the Tilt Series Setup of SerialEM.

To run Enable SerialEM option Tilt Series -> Run script at TS (on); then Tilt Series -> Set script to Run

```
MacroName DuringTomoHybridSta
# Written by Wim Hagen and Misha Kudryashev,
# MPI for Biophysics, May 2020

# Settings for exposure times and high-dose angle
HighExposure = 3
LowExposure = 0.25
TargetHighDoseAngle = 0

echo ################ DuringTomoHybridSta

SetUserSetting RefocusThreshold 1
SetTargetDefocus $TomoTargetDefocus
delay 5 sec

ReportTiltAngle
tiltangle = $RepVal1
anglediff = $TargetHighDoseAngle - $tiltangle
anglediff = ABS $anglediff
anglediff = ROUND $anglediff 1

If $anglediff < 0.1
  echo High dose exposure at start tilt!
  SetExposure R $HighExposure
  HighExposureFrameTime = $HighExposure / 40
  SetFrameTime  = $HighExposureFrameTime

ElseIf $anglediff > 0.1
  echo Normal dose exposure at other tilts.
  SetExposure R $LowExposure
  LowExposureFrameTime = $LowExposure / 10
  SetFrameTime  = $LowExposureFrameTime
Endif

KeepCameraSetChanges R
```

Supplementary Note 3

In this subsection we explain how to export a project from the Dynamo StA project to Relion. For that, purpose we will use the second, larger TMV dataset to recreate the results shown in Figures 3E and 3F. The original stacks, results of the Dynamo project and the necessary alignment files for the use in Imod can be downloaded from the EMPIAR database using the following link:
https://www.ebi.ac.uk/pdbe/emdb/empiar/entry/10393/
The files are the final Dynamo table file (.tbl); and the .st, .tlt, .xf and .defocus files for each tomogram. Below we will explain how to use the dyn2rel package, and then provide a description of it.

Preprocessing of input tomographic stacks:
Before aligning the tomographic stack, it must be normalized and weighted according to the applied electron dose. In our case, we want to maximize the dynamic range that can be represented in an unsigned 8-bit data type, that its, to have mean values $\mu = 128$, and standard deviations $\sigma_{LD} = 11$. As mentioned in the text of the manuscript, we used $e^{-}_{HD} = 15\ e^{-}/\text{Å}^2$ and $e^{-}_{LD} = 2\ e^{-}/\text{Å}^2$, the ratio of standard deviations is $\sigma_{HD} = 0.37 * \sigma_{LD} = 4$. This normalization is applied to each tomographic stack using the "newstack" command provided by the Imod package. In our dataset, the high-dose image was the 21-th projection on the stack:

```
newstack -in stack.st -ou stack_norm.st -meansd 128,11
newstack -in stack_norm.st -ou hd_frame.mrc -fr -se 21
newstack -in hd_frame.mrc -ou hd_frame_norm.mrc -meansd 128,4
newstack -in hd_frame_norm.mrc -ou stack_norm.st -fr -rep 21
```

Determining the defocus and assembling the Imod-style .defocus files
A simple way to detect the defocus per micrograph is by using the ctfplotter functionality from Etomo GUI (final aligned stack -> Correct Ctf). Ctfplotter can be scripted[27].

Alternatively, its possible to use Gctf which is fast and robust and works particularly well for untilted images. We use a Matlab script; please create a folder slices_forGctf and either copy the individual files there or create symbolic links according to the pattern

slices_forGctf/tomo_$N_proj_$M.mrc

where N is the running number of the tomogram, M – number of projection in a given tomogram. Go to the folder and run Gctf from your installation.

*Gctf-v1.06_sm_30_cu8.0_x86_64 *.mrc --apix 1.4 --kV 300 --defL 10000 --defH 80000*

Go to Matlab to your folder one above the slices_forGctf folder, run the following commands

```
list = dir('../*Tom*/tilt*.st'); % path to the imod folders

for tomo = 1:length(list)
   try
      stack_name = [list(tomo).folder '/' list(tomo).name];
      tlt_name   = [stack_name(1:end-3) '.tlt'];
      defocus_file_name = [stack_name(1:end-3) '.defocus'];
      tlt = textread(tlt_name);
      disp([tomo tlt(1)]);
```

```
    for i = 1:length(tlt)
       name_log = ['slices_forGctf/tomo_' num2str(tomo) '_proj_' num2str(i) '_gctf.log'];
       [status dfs] = system(['cat ' name_log ' | grep Final']);
       df_all(i) = (str2num(dfs(3:13)) + str2num(dfs(14:25)))/20;
    end

    text = [];
    text(1:length(tlt),1) = 1:length(tlt);
    text(1:length(tlt),2) = 1:length(tlt);
    text(1:length(tlt),3) = tlt;
    text(1:length(tlt),4) = tlt;
    text(1:length(tlt),5) = df_all(1:length(tlt));

    fid = fopen(['slices_forGctf/tomo' num2str(tomo) '.defocus'], 'wb');
    fprintf(fid, '%d\t %d\t %6.2f\t %6.2f\t %6.0f\t 2 \n', text(1,1:5) );
    for i = 2:length(tlt)
       fprintf(fid, '%d\t %d\t %6.2f\t %6.2f\t %6.0f \n', text(i,1:5) );
    end
    fclose(fid);
  catch
    disp(lasterr)
  end
end
```

You now have the .defocus files in the slices_forGctf folder, make sure that they make sense. If they do – exchange ['slices_forGctf/tomo' num2str(tomo) '.defocus'] to defocus_file_name in the fopen command and run the script again. Clean up the contents of the slices_forGctf folder.

After that tomographic reconstruction could be performed as usual. However, during tomographic processing, only use the rigid-body geometry for tomographic reconstruction. Do not use local alignments and offsets for tomogram positioning. After the tomographic reconstructions are performed, pick particles, perform subtomogram averaging. The current workflow assumes the Dynamo-style output.

Postprocessing with the hybrid StA script:

You can download the results of our conventional StA processing of the TMV dataset from Figure 3 from EMPIAR. In the dataset EMPIAR-10393 you can find all the files needed to export a Dynamo project into a Relion one. To simplify file access, we arranged the tomograms' files inside folders and named them following a simple naming convention. The downloaded folder should have the following scheme (showing the files for the first two tomograms only):

```
hybrid_tmv/
├── create_stack.m
├── data/
│   ├── tomo_01/
│   │   ├── tiltstack.defocus
│   │   ├── tiltstack.st
│   │   ├── tiltstack.tlt
│   │   └── tiltstack.xf
│   ├── tomo_02/
│   │   ├── tiltstack.defocus
│   │   ├── tiltstack.st
│   │   ├── tiltstack.tlt
│   │   └── tiltstack.xf
│   ├── tomo...
├── mask_b1.mrc
└── sta_rslt.tbl
```

In our case, we used the same name for each .st, .tlt, .ali and .defocus file inside a folder designated for each tomogram. This is not required, however helps to fill the required file name information in the dyn2rel.Tomogram class. The additional three files, "sta_rslt.tbl",

"mask_b1.mrc" and "create_stack.m", are the resulting table from the Dynamo project, the mask used in said project, and the main exporting script, respectively. The exporting procedure consists of three parts:

1. **Setting up tomograms:** The information of each tomogram is read into a *dyn2rel.Tomogram* class, and stored in an array of it. In our case we are using the original unbinned stack, with pixel size of 1.1 Å (recorded in superresolution mode on a K2). We must set up the .st, .xf, .tlt, and .defocus file for each tomogram, along with the full unbinned tomogram size, and the pixel size.
2. **Setting up the exporter:** A *dyn2rel.Exporter* is created and configured according to our Dynamo project. In our case, the table was the result of processing a once binned tomogram, so the coordinates and shifts in our table must be multiplied by 2 (exporter.tbl_mul = 2). Also, we want our Relion project to work with a box size of 200 voxels, but binned once too (bin=1). To accomplish this, we set up a cropping size of 400 and a binning of 1 (exporter.out_siz = 400; exporter.out_bin = 1). Finally, we used column 23 in our dynamo table to define to which particle half-set the particles belong, we provide this information for the exporter too (exporter.split_h = 23).
3. **Export the project:** The final step crops the particles from the stacks according to the information on the *dyn2rel.Tomogram* array and the table 'sta_rslt.tbl' and creates the MRCS stack and the STAR file. In our case, we chose the prefix 'hyb_b1' for the project. This creates the 'hyb_b1.mrcs' and 'hyb_b1.star' files.

The contents of the exporting script are:

```
% Contents of create_stack.m

%% SETTING UP TOMOGRAMS:

        % Tomogram indices used in sta_rslt.tbl.
        tomo_ix = [1 3 4 5];
        % as an alternative, we could use:
        % tbl = dread('sta_rslt.tbl');
        % tomo_ix = unique( tbl(:,20) );

        % Number of tomograms used in the project:
        N_TOMOS = 4;
        % alternatively we can use
        % N_TOMOS = length(tomo_ix);

        % Create the dyn2rel.Tomogram array with N_TOMO elements:
        tomos_list = dyn2rel.Tomogram.create_tomos_list(N_TOMOS);

        % All the tomograms have the same dimension and pixel size (unbinned).
        tomo_size = [7676, 7420, 1600];
        pix_size = 1.1;

        % Set up the information for each tomogram:
        for i = 1:N_TOMOS              % Set up filenames:
                base_tomo_dir = sprintf('data/tomo_%02d/',i);
                stack_name = [base_tomo_dir 'tiltstack.st'];
                tlt_name   = [base_tomo_dir 'tiltstack.tlt'];
                xf_name    = [base_tomo_dir 'tiltstack.xf'];
                def_name   = [base_tomo_dir 'tiltstack.defocus'];
```

```
        % Set the information for the i-th tomogram:
        tomos_list(i).index = tomo_ix(i);
        tomos_list(i).set_stack(stack_name,pix_size);
        tomos_list(i).read_ali_files(tlt_name,xf_name);
        tomos_list(i).set_tomogram_size(tomo_size);
        tomos_list(i).set_defocus( def_name );
        % optional:
        % tomos_list(i).HD_ix = 21;% the number of the high dose image in the seacks
    end

%% SETTING UP THE EXPORTER:

    exporter = dyn2rel.Exporter;

    % In our case, the DYNAMO project was run using binned data (half the size),
    % and the stack files (.st) are unbinned. Then, we have to use a factor of 2:
    exporter.tbl_mul = 2;

    % The StA map is a binned 200x200x200 volume, to have a hybrid map of the
    % same size, we need patches of 400 and then bin the data one time (according
    % to the DYNAMO's dbin command):
    exporter.out_siz = 400;
    exporter.out_bin = 1;

    % In our case, we stored the half-set information in the column 23 of the
    % table, so we pass that information to the exporter:
    exporter.split_h = 23;

    % Finally, we exclude 10% of the particles, the ones with the worst cross
    % correlation. Then, we set up xcor_sel to 90% of the particles:
    exporter.xcor_sel = 0.9;

    % Optionally, dependent on the handedness of the images, defocus could increase in
    % positive or negative direction along the height of the tomogram; this parameter can
    % flip the direction of the defocus; try both if not sure; this was not used for the TMV example
    % exporter.inv_dz = true;


%% EXPORT THE PROJECT:

    % Export table sta_rslt.tbl, using the tomograms in tomos_list, By giving
    % 'hyb_b1' as first argument, the method will create the files 'hyb_b1.star'
    % and 'hyb_b1.mrcs', which can be used with RELION:
    exporter.exec('hyb_b1',tomos_list,'sta_rslt.tbl');
```

Once the execution of the script is finished, we reconstruct the hybrid map to obtain the map from Figure 3E:

```
$ relion_reconstruct –-i hyb_b1.star –-o hyb_b1_rec.mrc --ctf --nr_helical_asu 15 --helical_rise 1.411626 --helical_twist 22.033413
```

or two half maps:

```
$ relion_reconstruct –-i hyb_b1.star –-o hyb_b1_h1.mrc --ctf --subset 1 --nr_helical_asu 15 --helical_rise 1.411626 --helical_twist 22.033413
$ relion_reconstruct –-i hyb_b1.star –-o hyb_b1_h2.mrc --ctf --subset 2 --nr_helical_asu 15 --helical_rise 1.411626 --helical_twist 22.033413
```

Finally, we refine the results to get the map from Figure 3F:

```
$ relion_refine –-i hyb_b1.star –-o Refine3D/run_001 --ctf --ref hyb_b1_rec.mrc --angpix 2.2 --o --flatten_solvent --solvent_mask mask_b1.mrc --firstiter_cc --ini_high 8 --offset_range 6 --offset_step 1 --ctf --split_random_halves --auto_refine --norm --scale --pool 4 --oversampling 1 --healpix_order 6 --sigma_rot 0.1 --sigma_tilt 0.05 --sigma_psi 0.05 --helix --helical_nr_asu 15 --helical_twist_initial 22.0337 --helical_twist_min 21.9 --helical_twist_max 22.1 --helical_twist_inistep 0.05 --helical_rise_initial 1.4123 --helical_rise_min 1.36 --helical_rise_max 1.46 --helical_rise_inistep 0.1 --helical_z_percentage 0.4 --helical_inner_diameter 20 --helical_outer_diameter 200 --helical_symmetry_search true --helical_keep_tilt_prior_fixed
```

This last command was executed on a computing cluster as a part of a submission script. In cases of poor alignment of tomograms or of subtomograms to the average, it is possible to attempt increasing --sigma_ang up to 0.2.

Finally, we exported the particles to cryoSPARC and performed per-particle CTF refinement (https://cryosparc.com/docs/tutorials/ctf-refinement). The resulting defocus values were used for another round of refinement in Relion which improved the resolution to 4.4 Å.

Note: For the RyR1 dataset we started with a traditional autorefine Relion project, however, in order to achieve higher resolution, we disabled the autorefinement and enabled the "always_cc" flag. The Relion's refinement command looks like this:

```
$ relion_refine --i autorefine_rslt.star --angpix 3.4 --o Refine3D/cc --sym c4 --iter 20 --flatten_solvent --ref autorefine_rslt.mrc --solvent_mask mask.mrc --ini_high 15 --offset_range 3 --offset_step 0.2 --ctf --norm --scale --pool 2 --oversampling 1 --healpix_order 7 --sigma_rot 0.1 --sigma_tilt 0.1--sigma_psi 0.1 --always_cc
```

Description of the dyn2rel package:

The Matlab package that perform the exporting procedure is called *dyn2rel*, and can be downloaded from the Kudryashev lab's GitHub repository (https://github.com/KudryashevLab/dyn2rel. To download the package, we go to the desired installation directory and use the *git* command. For this example, we installed the package in the "~/matlab_packages/dynamo_2_relion/" directory:

```
$ cd ~/matlab_packages/dynamo_2_relion/
$ git clone https://github.com/KudryashevLab/dyn2rel
```

To use the package in Matlab we have to add it to its path. In our current Matlab command line, we execute "addpath" and then "help" to validate the correct installation:

```
>> addpath ~/matlab_packages/dynamo_2_relion/
>> help dyn2rel
```

If the package was correctly installed, the last command will show the documentation of *dyn2rel*, which should list the main components of the package:

```
Contents of dyn2rel package:

    CTF - Holds the CTF's information.
    Exporter - Creates a RELION stack from a DYNAMO tbl and an Array of Tomograms.
    Tomogram - Holds a Tomogram's info: ST, XF, ALI, DEFOCUS files, and its size.
```

Here we give a brief description of the main classes and functions of the dyn2rel package:

- dyn2rel.CTF: (For internal usage mostly) class that holds the defocus information: U, V, angle, voltage, spherical aberration, amplitude contrast and Bfactor. Additional information can be read using the Matlab command:

```
>> help dyn2rel.CTF
```

- <u>dyn2rel.Exporter:</u> Class that exports the Dynamo project into Relion for 2D refinement. It reads the Dynamo table, projects the coordinates of each particle into the high-dose projection on the respective stack, crops the corresponding patch, stores it into a MRCS file and writes out a STAR file. The cropping procedure is controlled by the “xcor_sel” property, which sets the percentage of the best particles to be cropped (according to the cross correlation score, column 10 of a Dynamo table). The location of each particle is calculated by adding the shifts (columns 4, 5 and 6) to the position (columns 24, 25 and 26) and then multiplying the result by the “tbl_mul” property. The projection and defocus values are adjusted using the tomogram information (dyn2rel.Tomogram), and the cropped patch size is set by the “out_siz” property. After the patch is cropped, it can be inverted, according to the “invert” property, and binned, according to the “out_bin” property. Additionally, the “split_h” can be used to define how the random half sets are set. Here we give a small summary of the class’ properties grouped by purpose:
    - Particles selection:
        - xcor_sel: fraction of particles to be cropped, according to the cross correlation value (column 10 in a Dynamo table).
    - Table scaling:
        - tbl_mul: Multiplication factor to be applied to the position and shifts on the table to bring the table scaling to the unbinned tomogram scale.
    - Particle’s patch cropping:
        - out_siz: size of the unbinned cropped patch.
        - out_bin: Binning level of the patch. It is applied after the cropping stage, and it is done using Dynamo’s *dbin* command.
        - Invert: Is “true” the values on the patch will be inverted.
    - Extra information:
        - split_h: Sets how the value of “RandomSubset” will be set:
            - split_h < 1: the field will be not set.
            - split_h = 1: even-odd.
            - split_h > 1: the RandomSubset value will be set from the value of the “split_h”-th column in the Dynamo table. Column 23 of Dynamo tables is typically non-assigned.

Finally, the dyn2rel.Exporter class has only one method: exec. This method performs the exporting procedure. It requires 3 input parameters and produces one output:

- dyn2rel.Exporter.exec inputs:
    - out_pfx: (string) base name of the resulting files for the Relion project. The method creates a out_pfx.star file and a out_pfx.mrcs file.
    - tomo_list: (dyn2rel.Tomogram array) contains the information of the tomograms used in the project. It must be created with the dyn2rel.create_tomos_list function.
    - table: (Dynamo table, or filename) table created as a result of a Dynamo project.
- dyn2rel.Exporter.exec output:

- out_tbl: a subset of table input, containing only the particles that were exported.

For additional information, check the "help" of the class dy2rel.Exporter and its method:

```
>> help dyn2rel.Exporter
>> help dyn2rel.Exporter.exec
```

- dyn2rel.Tomogram: Class that holds the information of a Tomogram. That information includes unbinned tomogram size (tomo_size), file name of the stack (file_stack), defocus information and the projection information. To set up all the information, the "set_stack", "read_ali_files", "set_tomogram_size" and "set_defocus" methods must be used. Additionally, the "index" property must be set, and it is related to column 20 of a Dynamo table.
  This class also provides the static method "dyn2rel.Tomogram.create_tomos_list", which must be used to create a list of dyn2rel.Tomogram objects. This list is the one used by the dyn2rel.Exporter class.
  To see more information, check the help pages of the class:

```
>> help dyn2rel.Tomogram
>> help dyn2rel.Tomogram.set_stack
>> help dyn2rel.Tomogram.read_ali_files
>> help dyn2rel.Tomogram.set_tomogram_size
>> help dyn2rel.Tomogram.set_defocus
>> help dyn2rel.Tomogram.create_tomos_list
```
