# Supplementary material for: Subnanometer-resolution structure determination in situ by hybrid subtomogram averaging - single particle cryo-EM
Source: Nat Commun. 2020 Jul 24;11:3709. doi: 10.1038/s41467-020-17466-0 (PMC7381653; doi:10.1038/s41467-020-17466-0)
Supplement: Supplementary file 3 — Description of Additional Supplementary Information [file 41467_2020_17466_MOESM3_ESM.pdf]

Description of Additional Supplementary Files

File Name: Supplementary Movie 1

Description: Slices through a tomogram of native SR vesicles used in Figure 4 and Supplementary Figure 1. The width of the tomogram is 700 nm, it has been binned by a factor of four and subjected to ten iterations of non-linear anisotropic diffusion.
