# Supplementary material for: Subnanometer-resolution structure determination in situ by hybrid subtomogram averaging - single particle cryo-EM
Source: Nat Commun. 2020 Jul 24;11:3709. doi: 10.1038/s41467-020-17466-0 (PMC7381653; doi:10.1038/s41467-020-17466-0)
Supplement: Supplementary file 5 — Reporting Summary [file 41467_2020_17466_MOESM5_ESM.pdf]

nature research

Corresponding author(s): Mikhail Kudryashev

Last updated by author(s): Jun 8, 2020

Reporting Summary

Nature Research wishes to improve the reproducibility of the work that we publish. This form provides structure for consistency and transparency in reporting. For further information on Nature Research policies, see our Editorial Policies and the Editorial Policy Checklist.

Statistics

For all statistical analyses, confirm that the following items are present in the figure legend, table legend, main text, or Methods section.

n/a	Confirmed	
☐	☒	The exact sample size (*n*) for each experimental group/condition, given as a discrete number and unit of measurement
☐	☒	A statement on whether measurements were taken from distinct samples or whether the same sample was measured repeatedly
☒	☐	The statistical test(s) used AND whether they are one- or two-sided<br>*Only common tests should be described solely by name; describe more complex techniques in the Methods section.*
☒	☐	A description of all covariates tested
☒	☐	A description of any assumptions or corrections, such as tests of normality and adjustment for multiple comparisons
☒	☐	A full description of the statistical parameters including central tendency (e.g. means) or other basic estimates (e.g. regression coefficient) AND variation (e.g. standard deviation) or associated estimates of uncertainty (e.g. confidence intervals)
☒	☐	For null hypothesis testing, the test statistic (e.g. *F*, *t*, *r*) with confidence intervals, effect sizes, degrees of freedom and *P* value noted<br>*Give P values as exact values whenever suitable.*
☒	☐	For Bayesian analysis, information on the choice of priors and Markov chain Monte Carlo settings
☒	☐	For hierarchical and complex designs, identification of the appropriate level for tests and full reporting of outcomes
☒	☐	Estimates of effect sizes (e.g. Cohen's *d*, Pearson's *r*), indicating how they were calculated

Our web collection on statistics for biologists contains articles on many of the points above.

Software and code

Policy information about availability of computer code

Data collection	SerialEM
Data analysis	Imod Etomo, Dynamo, MotionCorr2, Gctf, Relion, CryoSPARC, Matlab

For manuscripts utilizing custom algorithms or software that are central to the research but not yet described in published literature, software must be made available to editors and reviewers. We strongly encourage code deposition in a community repository (e.g. GitHub). See the Nature Research guidelines for submitting code & software for further information.

Data

Policy information about availability of data

All manuscripts must include a data availability statement. This statement should provide the following information, where applicable:

- Accession codes, unique identifiers, or web links for publicly available datasets
- A list of figures that have associated raw data
- A description of any restrictions on data availability

The SerialEM scripts HybridDoseSymmetricTomo and DuringTomoHybridSta available in Supplementary notes 1 and 2. The processing workflow is implemented in MATLAB (Mathworks). The test hybrid dataset of TMV has been deposited to EMPIAR along with the alignment parameters for the tomograms, particle locations, and the alignment parameters for the particles in order to produce the hybrid map (EMPIAR-10393). The maps are deposited to EMDB: TMV hStA map- EMD – 10834, RyR1 hStA map - EMD-10840.
The documented scripts are presented in Supplementary note 3, the dyn2rel code is available on Github (https://github.com/KudryashevLab/dyn2rel). The tomographic tilt series of RyR1 will be released upon publication.

Field-specific reporting

Please select the one below that is the best fit for your research. If you are not sure, read the appropriate sections before making your selection.

☒ Life sciences ☐ Behavioural & social sciences ☐ Ecological, evolutionary & environmental sciences

For a reference copy of the document with all sections, see nature.com/documents/nr-reporting-summary-flat.pdf

Life sciences study design

All studies must disclose on these points even when the disclosure is negative.

Sample size	For structural analysis we attempted to maximize our sample size given the time on the limited microscope. The TMV datasets contained sufficient number of particles reinforced by symmetry, RyR1 dataset's resolution was limited by particle number which is a serious problem for many StA structures.
Data exclusions	NA
Replication	TMV datasets have been recorded in several sessions. RyR1 datasets have been shown consistent following the previous paper W Chen, M Kudryashev, EMBO reports 21 (5), e49891
Randomization	RYR1 particles were randomly distributed to the groups for independent processing; TMV particles were divided based on their Y-position in the tomograms.
Blinding	NA

Reporting for specific materials, systems and methods

We require information from authors about some types of materials, experimental systems and methods used in many studies. Here, indicate whether each material, system or method listed is relevant to your study. If you are not sure if a list item applies to your research, read the appropriate section before selecting a response.

Materials & experimental systems

n/a	Involved in the study	
☒	☐	Antibodies
☒	☐	Eukaryotic cell lines
☒	☐	Palaeontology and archaeology
☒	☐	Animals and other organisms
☒	☐	Human research participants
☒	☐	Clinical data
☒	☐	Dual use research of concern

Methods

n/a	Involved in the study	
☒	☐	ChIP-seq
☒	☐	Flow cytometry
☒	☐	MRI-based neuroimaging
